# Structural determinants at the M2 muscarinic receptor modulate the RGS4-GIRK response to pilocarpine by impairment of the receptor voltage sensitivity

**DOI:** 10.1038/s41598-017-05128-z

**Published:** 2017-07-21

**Authors:** I-Shan Chen, Kazuharu Furutani, Yoshihisa Kurachi

**Affiliations:** 1 Department of Pharmacology, Graduate School of Medicine, Osaka University, Suita, Osaka 565-0871 Japan; 2 Global Center for Medical Engineering and Informatics, Osaka University, Suita, Osaka 565-0871 Japan

## Abstract

Membrane potential controls the response of the M2 muscarinic receptor to its ligands. Membrane hyperpolarization increases response to the full agonist acetylcholine (ACh) while decreasing response to the partial agonist pilocarpine. We previously have demonstrated that the regulator of G-protein signaling (RGS) 4 protein discriminates between the voltage-dependent responses of ACh and pilocarpine; however, the underlying mechanism remains unclear. Here we show that RGS4 is involved in the voltage-dependent behavior of the M2 muscarinic receptor-mediated signaling in response to pilocarpine. Additionally we revealed structural determinants on the M2 muscarinic receptor underlying the voltage-dependent response. By electrophysiological recording in *Xenopus* oocytes expressing M2 muscarinic receptor and G-protein-gated inwardly rectifying K^+^ channels, we quantified voltage-dependent desensitization of pilocarpine-induced current in the presence or absence of RGS4. Hyperpolarization-induced desensitization of the current required for RGS4, also depended on pilocarpine concentration. Mutations of charged residues in the aspartic acid-arginine-tyrosine motif of the M2 muscarinic receptor, but not intracellular loop 3, significantly impaired the voltage-dependence of RGS4 function. Thus, our results demonstrated that voltage-dependence of RGS4 modulation is derived from the M2 muscarinic receptor. These results provide novel insights into how membrane potential impacts G-protein signaling by modulating GPCR communication with downstream effectors.

## Introduction

Membrane potential controls physiological events through regulating a variety of protein functions, including modulation of ligand binding and signal transduction in G-protein-coupled receptors (GPCRs)^1–5^. The M2 muscarinic receptor is the one of the most intensively studied GPCRs in its voltage-dependent behaviors and molecular mechanisms^1–4, 6–12^. For example, membrane hyperpolarization increases the affinity of the M2 muscarinic receptor for its native full agonist, acetylcholine (ACh), and decreases affinity for its partial agonist pilocarpine^3, 9, 10^. Conversely, pilocarpine binding enhances the voltage-induced conformational change of the M2 muscarinic receptor, while ACh binding immobilizes the charge movement^4^. Therefore, access to some conformational states of the M2 muscarinic receptor is thought to be dependent on the binding of specific agonists, and membrane potential modulates the transition between these conformational states^2, 10, 11, 13^. However, how the voltage-dependent states stabilized by different agonists regulate cellular responses is not well understood.

One of the important regions for the voltage-dependent behavior of M2 muscarinic receptor toward its agonist is intracellular loop 3, which is also the key motif responsible for G-protein interaction^3, 12, 14, 15^. Mutation of amino acid residues KKDKK located in the N-terminal region of intracellular loop 3 to the sequence ELAAL abolish the voltage-dependence of ACh binding^3, 12^. The aspartic acid-arginine-tyrosine (DRY) motif of the M2 muscarinic receptor could also be an important region for its voltage-sensitivity^3^. The DRY motif is critical for regulating GPCR conformational states and agonist-induced response^6, 14, 16, 17^. Neutralization of D120^3.49^ and R121^3.50^ (D120N/R121N mutant) in the DRY motif has been proposed to abolish the charge movement of the M2 muscarinic receptor^3^. However, another study concluded that gating current still occurs in the D120N/R121N mutant and therefore these residues are not the key motif responsible for voltage sensor^4^. Therefore, it remains controversial whether the DRY motif acts as a voltage sensor or not^1, 7^. There is also no evidence whether the voltage sensor function of the M2 muscarinic receptor via DRY motif or the intracellular loop 3 can regulate its downstream G-protein signaling regulators.

We previously revealed that the regulator of G-protein signaling (RGS) 4 protein modulates the cellular efficacy of pilocarpine^18^. RGS proteins are known to negatively regulate G-protein signaling by accelerating GTP hydrolysis to GDP by G_α_ subunit and thereby terminating the G-protein signal^19–23^. Pilocarpine promotes the RGS4-mediated inhibition of M2 muscarinic receptor-activated G-protein signaling, leading to a smaller G-protein-gated inwardly rectifying K^+^ (GIRK) current than that of ACh^18^. Importantly, membrane potential controls this phenomenon^18^. However, how RGS4 is involved in the voltage-dependent behavior of M2 muscarinic receptor**-**activated signaling remains unclear.

Since RGS4 is not a membrane protein, RGS4 seems unlikely to function as a voltage sensor. This suggests the voltage sensitivity of RGS4 is derived from its coupled membrane protein, M2 muscarinic receptor. If so, the M2 muscarinic receptor would act as a voltage sensor not only toward its agonists but also toward the RGS4. The DRY motif and the intracellular loop 3 at the M2 muscarinic receptor are candidates for the structural determinants which contribute to the voltage-dependence of RGS4.

Here, we characterized the voltage-dependence of RGS4 modulation on M2 muscarinic receptor-activated signaling and revealed which receptor motif contributes to the underlying mechanism. With electrophysiological recordings from *Xenopus* oocytes, we demonstrate that RGS4 triggers a voltage-dependent current decay of pilocarpine-induced GIRK current. By structure-function analysis of the DRY motif and the intracellular loop 3 of M2 muscarinic receptor using mutagenesis methods, we observed that mutations of charged residues in the DRY motif (D120^3.49^ and R121^3.50^), instead of intracellular loop 3, significantly impaired the voltage-dependence of RGS4 modulation. The results demonstrate a novel mechanism that membrane potential modulates RGS4 function through the voltage-sensitive M2 muscarinic receptor.

## Results

### RGS4 modulation of M2 muscarinic receptor-mediated GIRK currents by pilocarpine is voltage-dependent

To understand the effects of membrane potential on RGS4 function, we recorded the M2 muscarinic receptor-activated GIRK currents in rat atrial myocytes and *Xenopus* oocytes heterologously expressing the M2 muscarinic receptor, a cardiac-type GIRK channel (Kir3.1/Kir3.4 heterotetramer^24^) and RGS4^25, 26^.

**Figure 1:**
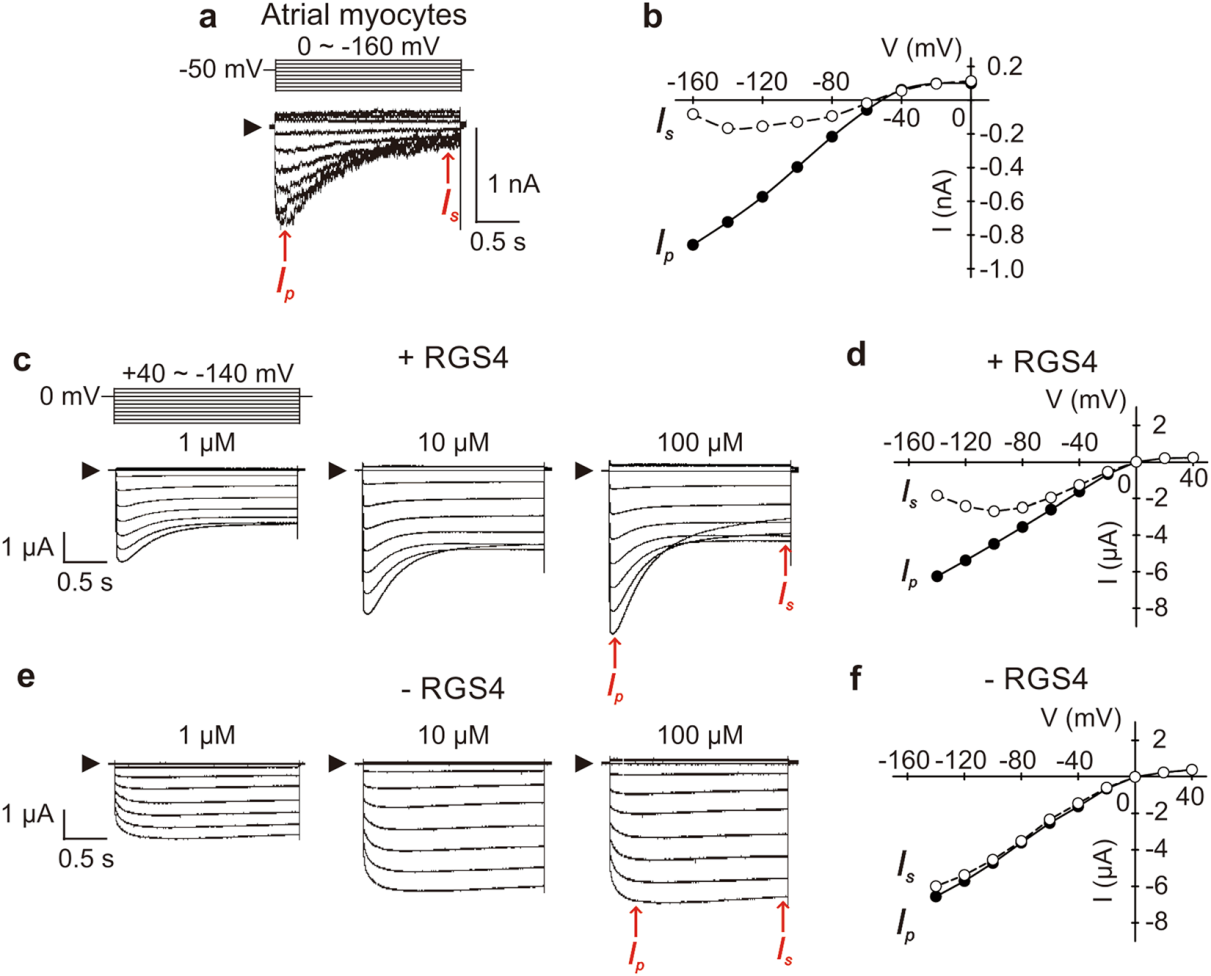
Voltage-dependent response of pilocarpine-induced GIRK currents in rat atrial myocytes and *Xenopus* oocytes. (**a**) Pilocarpine (100 µM)-induced GIRK currents in rat atrial myocytes were recorded with a step pulse protocol as shown above the current traces. Basal currents were subtracted. (**b**) I-V curve of pilocarpine-induced GIRK currents in atrial myocytes. *I*_p_ (filled circles) indicates the peak current at an activated state of GIRK channel; *I*_s_ (open circles) indicates the steady-state current at the end of the voltage pulse. Red arrows in **a** were used to reconstruct the I-V curve in **b**. (**c**–**f**) Pilocarpine (1, 10, 100 μM)-induced GIRK currents in *Xenopus* oocytes expressing M2 muscarinic receptor, Kir3.1/Kir3.4 with (**c**,**d**) and without (**e**,**f**) RGS4 were recorded with a step pulse protocol shown above. Basal currents were subtracted. Amplitudes of *I*_p_ and *I*_s_ indicated by red arrows in **c** and **e** were used to reconstruct the I-V curve in **d** and **f**.

In atrial myocytes (Fig. 1a,b) and oocytes expressing RGS4 (Fig. 1c,d), pilocarpine-induced GIRK currents during membrane hyperpolarization reached a peak (*I*_p_) and then decreased to steady state by pulse-end (*I*_s_). This current decay suggests a desensitization of GIRK currents to pilocarpine at hyperpolarized potential in the presence of RGS4. The tau of current decay in the presence of 100 µM pilocarpine at –140 mV in RGS4-expressing oocytes was 0.47 ± 0.02 s (n = 6). This phenomenon was found to be due to the cessation of G-protein activation by modulation of GTP hydrolysis via RGS4 in our previous study using GTPγS^18^, a non-hydrolyzable analog of GTP. The current decay during hyperpolarization was not observed in the absence of RGS4 (Fig. 1e,f), suggesting that RGS4 is a crucial mediator in the hyperpolarization-induced desensitization of the GIRK channel. This suggests that the mechanism of desensitization is an RGS4-mediated reduction in active G-proteins. Note that the term ‘desensitization’ does not imply an insensitivity of the GIRK channel itself.

In oocytes expressing RGS4, with 100 µM pilocarpine, *I*_s_ during –140 mV pulses was smaller than *I*_s_ at more positive potentials (Fig. 1c). However, the current-voltage (I-V) curve of *I*_p_ was linear at hyperpolarized potentials, indicating GIRK currents are not yet desensitized immediately after hyperpolarization (Fig. 1d). The I-V curve of *I*_s_ showed a different shape from that of *I*_p_. The differences in current amplitude between *I*_p_ and *I*_s_ indicate that desensitization increases as the membrane potential become more hyperpolarized (Fig. 1d). This phenomenon was not observed in the absence of RGS4 (Fig. 1e,f). The results demonstrate that hyperpolarization acts through RGS4 to desensitize GIRK channels to pilocarpine.

**Figure 2:**
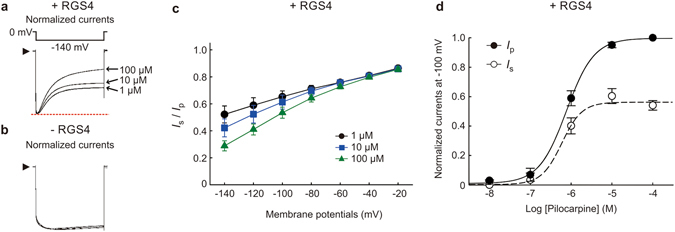
Effects of RGS4 on modulation of GIRK current in a pilocarpine concentration-dependent manner. (**a**,**b**) Pilocarpine (1, 10, 100 μM)-induced GIRK currents in *Xenopus* oocytes expressing M2 muscarinic receptor, Kir3.1/Kir3.4 with (**a**) and without (**b**) RGS4 were recorded at −140 mV with the test pulse protocol shown above. The *I*_p_ evoked by different concentrations of pilocarpine (1, 10, 100 μM) were normalized to the same level (red dashed line). Different levels of *I*_s_ in the presence of different pilocarpine concentrations were observed. (**c**) The ratio of *I*_s_ to *I*_p_ (*I*_s_/*I*_p_) at hyperpolarized potentials (−20 mV to −140 mV) in the applications of pilocarpine (1 µM, black circles; 10 µM, blue squares; 100 µM, green triangles) in oocytes expressing RGS4. Data are means ± s.e.m., n = 6–10. (**d**) Concentration-response curves of *I*_s_ (filled circles) and *I*_p_ (open circles) of pilocarpine-induced GIRK current in the presence of RGS4 were fitted with the Hill equation. The maximal value of *I*_p_ of pilocarpine-induced GIRK current was normalized to 1. The –logEC_50_ was 6.23 ± 0.12 M for *I*_s_; 6.11 ± 0.02 M for *I*_p_. Data are means ± s.e.m., n = 3–6.

We next normalized the GIRK currents induced by different concentrations of pilocarpine with the same amplitude of *I*_p_ and then compared the level of *I*_s_ in the presence and absence of RGS4 (Fig. 2a,b). With RGS4, we observed that a higher concentration (100 μM) of pilocarpine had more impact on the normalized *I*_s_ level than a lower concentration (1 μM). The ratio *I*_s_/*I*_p_ decreased at high concentrations of pilocarpine and hyperpolarized potentials in the presence of RGS4 (Fig. 2c), suggesting that RGS4 inhibits GIRK currents in a pilocarpine concentration-dependent manner. We then fitted the concentration-response curve of *I*_s_ and *I*_p_ at −100 mV in the presence of RGS4 with the Hill equation (Fig. 2d). There was no considerable difference in the −logEC_50_ of *I*_s_ and *I*_p_ (−logEC_50_ was 6.23 ± 0.12 M for *I*_s_; 6.11 ± 0.02 M for *I*_p_), indicating that the receptor affinity of pilocarpine was not substantially changed by the presence of RGS4 modulation. We observed the maximal inhibitory effect of RGS4 modulation in the presence of saturated concentration of pilocarpine (100 μM). These results suggest that RGS4-GIRK response is voltage-dependent, and this depends on pilocarpine binding to the M2 muscarinic receptor.

### Membrane potential rapidly modulates RGS4-mediated desensitization of M2 muscarinic receptor-GIRK signaling

**Figure 3:**
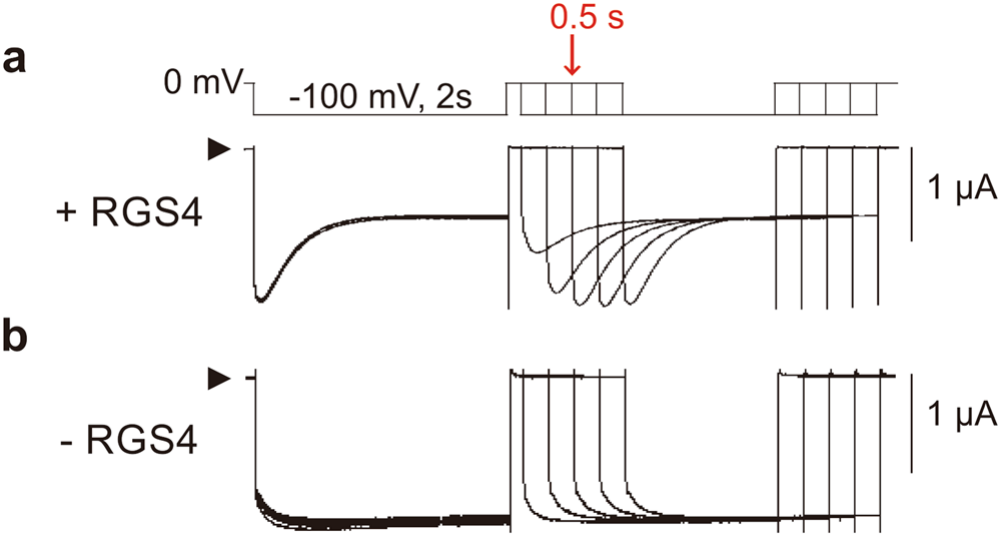
Effects of RGS4 on the recovery of GIRK currents from desensitization. (**a**,**b**) Pilocarpine (100 µM)-induced GIRK currents in oocytes expressing M2 muscarinic receptor, Kir3.1/Kir3.4 with (**a**) and without (**b**) RGS4 were recorded with a protocol shown above. Current were recorded with two hyperpolarizing pulses from 0 mV to −100 mV for 2 s which were separated by an interpulse of 0 mV for a duration range of 0.1, 0.3, 0.5, 0.7 and 0.9 s. The fully recovery of GIRK current from the desensitized state can be observed when interpulse (0 mV) duration is longer than 0.5 s. Time constant for the recovery was fitted with exponential curve and discussed in the result section.

We next characterized the kinetics of the voltage-dependent response. The time course of GIRK current recovery from desensitization was examined with two hyperpolarizing pulses protocol (−100 mV for 2 s) which were separated by an interpulse (0 mV) of different durations (0.1, 0.3, 0.5, 0.7 and 0.9 s) (Fig. 3). Compared with the first hyperpolarizing pulse in the presence of RGS4, the pilocarpine-induced current showed a smaller *I*_p_ at the second hyperpolarizing pulse when the interpulse duration was 0.1 s (Fig. 3a). Prolonging the duration of interpulse to 0.5 s recovered the current amplitude of *I*_p_. The tau of *I*_p_ recovery was 0.16 ± 0.01 s (n = 6) by exponential curve fitting. We did not observe hyperpolarization-induced desensitization in the absence of RGS4 (Fig. 3b). The results demonstrate that the M2 muscarinic receptor-GIRK signaling recovers from hyperpolarization-induced RGS4-mediated desensitization very rapidly.

### Mutations in the DRY motif of the M2 muscarinic receptor, instead of intracellular loop3, impaired RGS4 modulation of GIRK current mediated by pilocarpine

**Figure 4:**
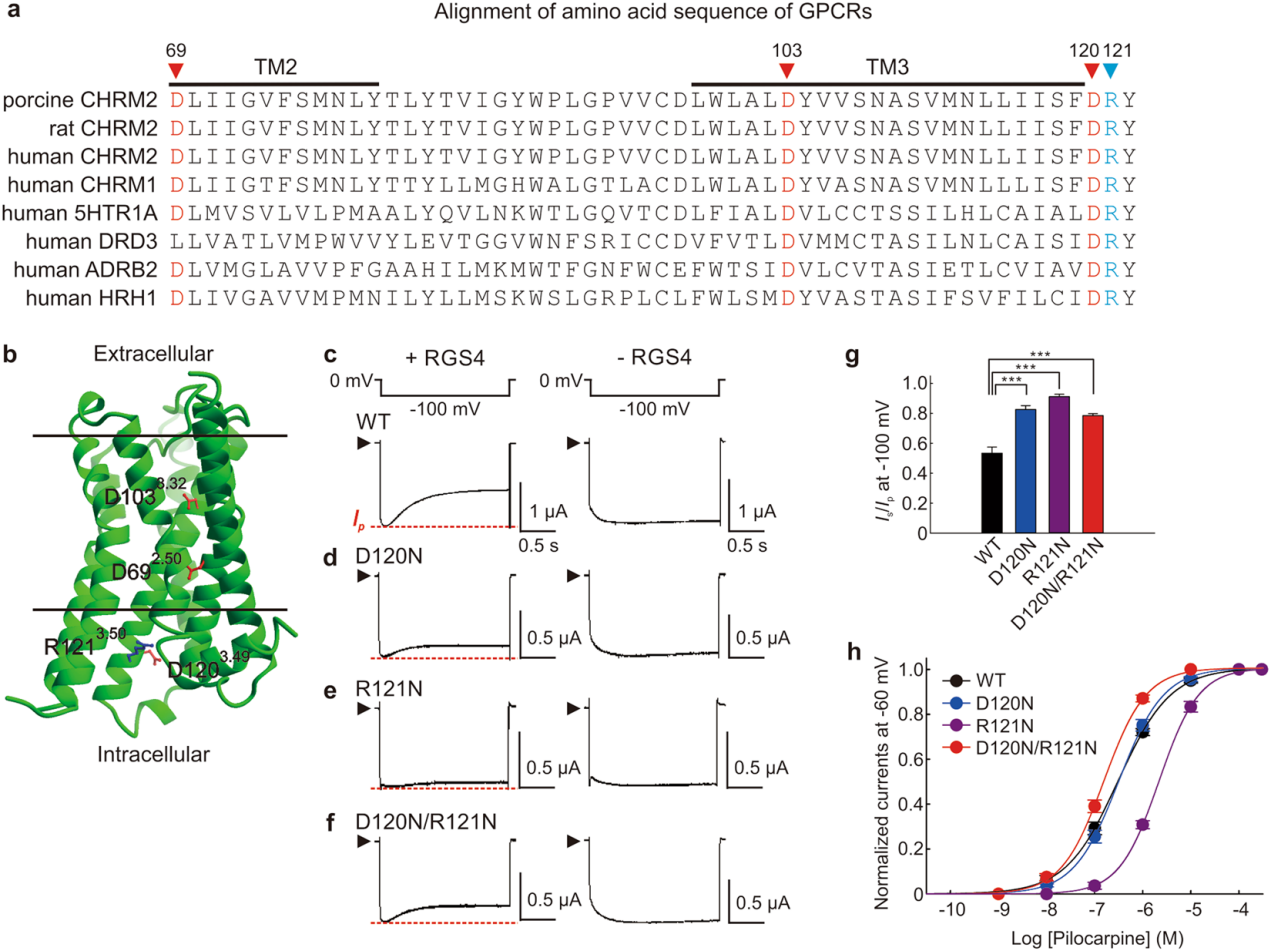
Charged residues in the DRY motif of the M2 muscarinic receptor contribute to the voltage-dependent response of GIRK currents. (**a**) Alignment of amino acids of different species of M2 muscarinic receptors and other human rhodopsin-like GPCRs. Triangles indicate the position of charged residues D69^2.50^, D103^3.32^, D120^3.49^ and R121^3.50^. (**b**) M2 muscarinic receptor model was based on the crystal structure (PDB accession 4MQS)^27^. The charged residues that we investigated in this study are colored. (**c**–**f**) Pilocarpine (100 µM)-induced GIRK currents in oocytes expressing the WT M2 muscarinic receptor (**c**) or mutants D120N (**d**), R121N (**e**) and D120N/R121N (**f**) with (*left panels*) and without (*right panels*) RGS4 were recorded at −100 mV as shown above. (**g**) The ratio of *I*_s_ to *I*_p_ (*I*_s_/*I*_p_) of pilocarpine-induced GIRK current at −100 mV in oocytes expressing M2 muscarinic receptor WT and mutants. (**h**) Concentration-response curves of pilocarpine-induced GIRK current were fitted with the Hill equation. The maximal value of pilocarpine-induced GIRK current was normalized to 1. The −log EC_50_ was 6.51 ± 0.01 for WT; 6.81 ± 0.01 for D120N; 5.66 ± 0.01 for R121N; 6.50 ± 0.01 for D120N/R121N. Data in g and h are means ± s.e.m., n = 6–8. ***Indicates a statistically significant difference (*P* ≤ 0.001).

To reveal the mechanism which underlies the voltage-dependence of RGS4 modulation, we focused on the voltage-sensitive M2 muscarinic receptor. We examined several charged amino acid residues of the M2 muscarinic receptor which are highly conserved in the rhodopsin-like GPCR family and may act as a voltage-sensor (Fig. 4a).

We first mutated charged residues D69^2.50^ and D103^3.32^ (Fig. 4a,b), are in the transmembrane domains (TMs) of M2 muscarinic receptor and are relevant to voltage sensing and receptor activation^4, 27, 28^. Consistent with previous studies, we found that mutation of these residues to alanine (D69A and D103A) or asparagine (D69N and D103N) abolished ACh- and pilocarpine-induced GIRK currents in oocytes (Supplementary Fig. S1). This change may be due to the impairment of ligand binding in the mutation of D103^3.32^ or the reduced protein surface expression in the mutation of D69^2.50^ ^4, 27^.

We next mutated charged residues D120^3.49^ and R121^3.50^ located in the DRY motif of the M2 muscarinic receptor (Fig. 4a,b). Mutations that neutralized the charges of D120^3.49^ (D120N) or R121^3.50^ (R121N) singly, or both residues together (D120N/R121N), decreased the amplitude difference between the *I*_p_ and the *I*_s_ of pilocarpine-evoked GIRK currents at −100 mV (Fig. 4d–f), as compared with that of wild-type (WT) M2 muscarinic receptor (Fig. 4c) in the presence of RGS4. During a hyperpolarization pulse, the currents of oocytes expressing these three mutants only showed a modest decrease from *I*_p_ to *I*_s_ in the presence of RGS4 (Fig. 4d–f, *left panel*). Such current decay during hyperpolarization was not seen in the absence of RGS4 (Fig. 4c–f, *right panel*). The ratios *I*_s_/*I*_p_ of the M2 muscarinic receptor mutants were significantly elevated compared with WT M2 muscarinic receptor (0.83 ± 0.03 for D120N; 0.91 ± 0.02 for R121N; 0.79 ± 0.01 for D120N/R121N; 0.53 ± 0.04 for WT, *P* ≤ 0.001 for each mutant when compared with WT) (Fig. 4g). This suggests that neutralization of D120^3.49^ or R121^3.50^ diminishes the hyperpolarization-induced desensitization of GIRK current by RGS4. To confirm whether mutations of D120^3.49^ and R121^3.50^ affect the pilocarpine-induced current, we calculated the concentration-response curves of these mutants. The concentration-response curves of D120N for pilocarpine- (Fig. 4h and Supplementary Fig. S2) and ACh-induced GIRK currents (Supplementary Fig. S3) were similar with WT, while the concentration-response curves of R121N for both pilocarpine and ACh were a rightward shift from that of WT. The 100 µM concentration of pilocarpine saturated current in all DRY mutants we tested. Although, as we shown in Fig. 2, the pilocarpine-bound M2 muscarinic receptor enhances RGS4-mediated desensitization of GIRK response at hyperpolarized potentials, the DRY motif mutants impaired this function. Therefore, the DRY motif of the M2 muscarinic receptor is an essential structural determinant of the voltage sensitivity of RGS4.

**Figure 5:**
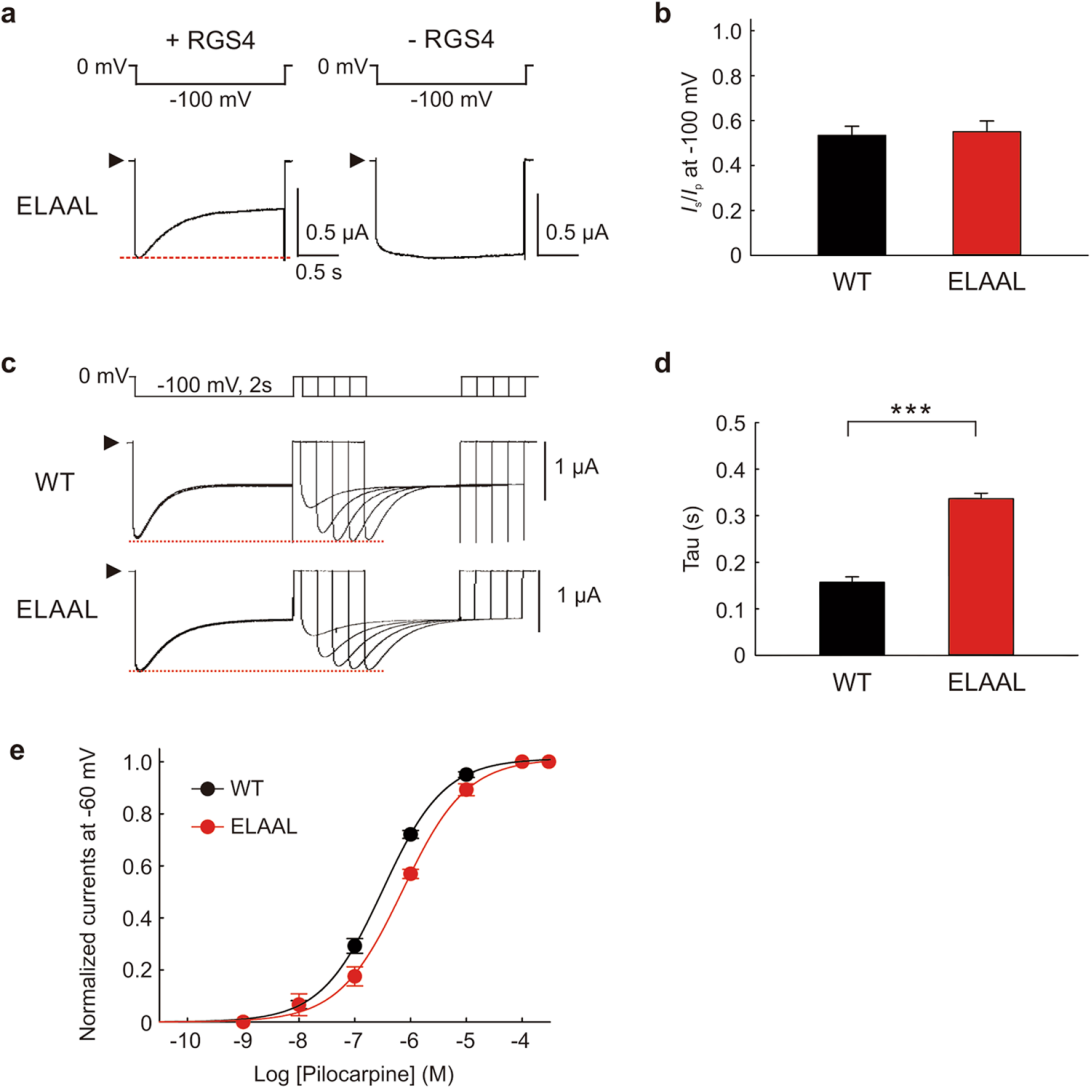
Effects of mutations in the intracellular loop 3 of M2 muscarinic receptor on the voltage-dependent response of GIRK currents. (**a**) Pilocarpine (100 µM)-induced GIRK currents in oocytes expressing M2 muscarinic receptor mutant ELAAL with (*left panels*) and without (*right panels*) RGS4 were recorded at −100 mV as shown above. (**b**) The ratio of *I*_s_ to *I*_p_ (*I*_s_/*I*_p_) of M2 muscarinic receptor WT and ELAAL at –100 mV in the presence of pilocarpine (100 µM). (**c**) Recoveries of pilocarpine (100 µM)-induced GIRK currents from hyperpolarization-mediated desensitization of channels in oocytes expressing WT and ELAAL M2 muscarinic receptors were recorded with a protocol shown above the current traces. (**d**) The time constant (tau) of *I*_p_ recovery times in **c** were calculated with exponential curve fitting. (**e**) Concentration-response curves of pilocarpine-induced GIRK currents were fitted with the Hill equation. The maximal value of pilocarpine-induced GIRK current was normalized to 1. The −logEC_50_ was 6.51 ± 0.02 for WT; 6.15 ± 0.02 for ELAAL. Data in **b**, **d** and **e** are means ± s.e.m., n = 6. ***Indicates a statistically significant difference (*P* < 0.001).

Since the intracellular loop 3 of the M2 muscarinic receptor is also involved in the voltage-dependence of ACh binding^3^, we examine the roles of the intracellular loop 3 in the voltage-dependence of RGS4 modulation of pilocarpine-induced GIRK current. Interestingly, we found that, in our oocyte expression system, the M2 muscarinic receptor ELAAL mutant retained voltage-dependent response of pilocarpine-induced GIRK currents in the presence of RGS4 (Fig. 5a). The current of ELAAL mutant showed normal hyperpolarization-induced current decay in the presence of RGS4. The *I*_s_/*I*_p_ of ELAAL mutant (0.55 ± 0.05, n = 6) was similar to that of WT M2 muscarinic receptor (0.53 ± 0.04, n = 6) (Fig. 5b), suggesting that the N-terminal region of intracellular loop 3 is not essential for the voltage-dependence of RGS4 modulation. The current of ELAAL mutant fully recovered *I*_p_ from the desensitization when the interpulse duration was 0.9 s, longer than with the WT M2 muscarinic receptor (Fig. 5c). The tau for *I*_p_ recovery was 0.16 ± 0.01 s for WT and 0.34 ± 0.01 s for ELAAL, respectively (n = 6 for each, *P* < 0.001 in Fig. 5d). The slow *I*_p_ recovery of ELAAL mutant from the hyperpolarization-induced desensitization suggests that this mutant takes a longer time to recover the GIRK current from RGS4 modulation as compared with WT. The *I*_s_/*I*_p_ was, however, not influenced by mutation of KKDKK to ELAAL (Fig. 5b). Concentration-response curves of ELAAL mutant for pilocarpine (Fig. 5e and Supplementary Fig. S2) and ACh (Supplementary Fig. S3) were slightly rightward shifted as compared with that of WT. Taken together, the results indicate that the N-terminal region of intracellular loop 3 is not essential for voltage-dependence of RGS4.

## Discussion

In the present study, we find that membrane potential modulates RGS4 function through the voltage-sensitive M2 muscarinic receptor. Hyperpolarization enhanced the RGS4-mediated inhibition of pilocarpine-induced GIRK currents in both rat atrial myocytes and *Xenopus* oocytes (Fig. 1). This phenomenon is dependent on the pilocarpine concentration (Figs 1 and 2). A previous study suggested that hyperpolarization induces a decrease in M2 muscarinic receptor affinity toward pilocarpine in cat atrial myocytes^9^. However, in the absence of RGS4, pilocarpine-induced GIRK current only showed a negligible decrease in current amplitude during hyperpolarization. Therefore, the hyperpolarization-induced desensitization of GIRK channel appears to be due to the increase in the capability of RGS4 modulation, not a decrease in ligand affinity.

Analyses of gating charge-movement and reporter fluorescent signal associated with M2 muscarinic receptor have suggested that a voltage-induced conformational change in M2 muscarinic receptor occurs very rapidly, within 0.01 s^3, 4, 12^. This would precede the voltage-induced changes in RGS4 function. Our results showed that RGS4-mediated desensitization of GIRK currents recovers rapidly (tau of recovery time was about 0.16 s) at holding membrane potential (Fig. 3), and this event would follow the receptor conformational change mediated by voltage change.

We showed that D120^3.49^ or R121^3.50^ in the DRY motif of M2 muscarinic receptor contribute to the voltage-dependence of RGS4 modulation of GIRK current. These charged residues are part of the underlying mechanism that enhances the capability of RGS4 modulation at hyperpolarized potentials (Fig. 4). This result indicates that the voltage-dependence of RGS4 is derived from the M2 muscarinic receptor. One possible mode of action for D120^3.49^ and R121^3.50^ is that these residues act as part of voltage sensors in the receptor for sensing membrane potential. Another possible mode is that these residues are required for its interaction with RGS4 because DRY motif is located on the cytosolic side of the plasma membrane. However, it is most likely that these charged residues support the pathway that mediates voltage-dependent conformational change of the M2 muscarinic receptor because these charged residues form a part of charge-charge interaction which stabilizes the receptor conformation^14^. These charged residues respectively interact with other residues located in the TM2 and TM6 as well^14, 16, 27^. Once the negative charge of D120^3.49^ is neutralized, the converse R121^3.50^ is probably free to move to find another ionic partner^29^ and vice versa. Therefore, neutralization of both D120^3.49^ and R121^3.50^ or only one of them disturbs the balance of charge-charge interaction within receptor and thus altering the voltage-sensitivity.

Other charged residues of the M2 muscarinic receptor are relevant to the voltage sensing, including D69^2.50^ and D103^3.32^ ^4^. However, mutations of D69^2.50^ or D103^3.32^ appear to reduce both receptor expression and agonist binding^4, 27, 28^. These changes may be the reason why we could not observe agonist-induced currents and limits our evaluation of their roles to the present experimental design.

Membrane potential regulates the binding and dissociation of ACh with the M2 muscarinic receptor through the N-terminal region of the intracellular loop 3^3, 12, 30^. In the present study, we showed that replacing the N-terminal region of the intracellular loop 3 of the M2 muscarinic receptor (residues KKDKK) to ELAAL decelerates the recovery of the voltage-induced change in pilocarpine-induced GIRK current as compared with WT in the presence of RGS4 (Fig. 5). Several studies have demonstrated that replacing intracellular loop 3 of the M2 muscarinic receptor with the corresponding sequence from the M1 muscarinic receptor or β_1_-adrenergic receptor changes the specificity for different subtypes of G_α_ and changes the ligand-mediated activation of G-protein signaling^31–33^. Therefore, the affinity of the ELAAL mutant for the G_i/o_ type of G_α_ should be different from WT M2 muscarinic receptor. The G_i/o_-RGS4 complex may be associated with intracellular loop 3 of the ELAAL mutant in a less stable state, as compared with WT, and thus decelerates the recovery of G-protein signaling from RGS4-mediated regulation.

Recent studies demonstrated that the voltage-dependence of the M1 muscarinic receptor toward ACh binding is opposite to the M2 muscarinic receptor^2^ and replacing intracellular loop 3 of the M2 muscarinic receptor with that of M1 muscarinic receptor gives the mutated receptor the voltage-dependence of the M1 muscarinic receptor^3^. However, our results demonstrated that the ELAAL mutant retains the voltage-dependence of RGS4 function. This suggests that this region is responsible for the voltage-regulated coupling of the GPCR to its G-protein^30^, and this pathway is independent to the voltage-dependence of RGS4 modulation on G-protein signal. Taken together, both the positive pathway (agonist-induced activation) and the negative pathway (RGS4-mediated inhibition) of G-protein signaling are controlled by voltage via different motif at M2 muscarinic receptor, and thus modulating the receptor communication with downstream effectors, G_i/o_ and RGS4.

As several RGS-associated GPCRs have been identified^34–37^, further research will be required to fully understand the interaction between the receptor and RGS protein, which underlies the voltage-dependent modulation of RGS in regulating downstream GPCR signaling. The C-terminal tail of GPCR proteins is a candidate region for mediating the association between RGS proteins and GPCRs^38, 39^.

In the present study, we showed that membrane potential modulates RGS4 function through the voltage-sensitive M2 muscarinic receptor. Especially at hyperpolarized potentials, pilocarpine, which categorized as a “partial agonist” may, in fact, stabilize a receptor conformation which leads to relatively high GTP hydrolysis activity as compared to that of a full agonist. This study sheds light on the multimodal functions of the M2 muscarinic receptor in both signal perception and transduction.

## Methods

### Animals and cell preparations

Animal experiments were performed in accordance with the guidelines for the use of laboratory animals of Osaka University Graduate School of Medicine. The experimental protocol was approved by the Institutional Animal Care and Use Committee and the Animal Experiments Committee of Osaka University. Single atrial myocytes were enzymatically isolated from hearts of adult male Wistar rats (200–300 g) as previously described^40^ and maintained in KB solution containing 10 mM taurine, 10 mM oxalic acid, 70 mM glutamic acid, 25 mM KCl, 10 mM KH_2_PO_4_, 0.5 mM EGTA, 11 mM glucose and 10 mM HEPES (pH 7.3 with KOH, 295–300 mOsm L^−1^). Freshly isolated cells were used in patch clamp experiments on the day.

The cRNA of porcine M2 muscarinic receptor (80 ng oocyte^−1^), mouse Kir3.1 (8 ng oocyte^−1^), rat Kir3.4 (8 ng oocyte^−1^) and rat RGS4 (160 ng oocyte^−1^) were used in this study. The porcine M2 muscarinic receptor was encoded on pSP64T plasmid. Point mutations in the M2 muscarinic receptor were constructed by PCR mutagenesis and verified by sequencing. The positions of amino acid residues of M2 muscarinic receptor are indicated by their generic number according to the Ballesteros–Weinstein nomenclature^41^. Complementary RNAs were transcribed from the cDNA by mMessage mMachine kit (Ambion). *Xenopus laevis* oocytes were prepared and injected with cRNA as described previously^42^. After injection, oocytes were incubated at 18 °C in ND96 solution containing 96 mM NaCl, 2 mM KCl, 1.8 mM CaCl_2_, 1 mM MgCl_2_ and 5 mM HEPES (pH 7.6 with NaOH) and supplemented with gentamicin (50 µg ml^−1^). Currents were recorded 3–5 days after the cRNA injection.

### Electrophysiological recording

Voltage-clamped currents of atrial myocytes were recorded in the whole-cell configuration by a patch-clamp amplifier (EPC10, HEKA Electronics, Lambrecht, Germany) at room temperature. Data were low-pass-filtered at 1 kHz (–3 dB) by an eight-pole Bessel filter, sampled at 5 kHz, and analyzed offline with PatchMaster (HEKA Electronics) and FitMaster (HEKA Electronics). Myocytes were recorded in a bath solution containing 115 mM NaCl, 20 mM KCl, 1.8 mM CaCl_2_, 0.53 mM MgCl_2_, 5.5 mM glucose and 5.5 mM HEPES (pH 7.4 with NaOH). The tip resistance of glass electrodes was 2–5 MΩ when filled with the pipette solution containing 150 mM KCl, 5 mM EGTA, 1 mM MgCl_2_, 3 mM K_2_ATP, 0.1 mM Na_2_GTP and 5 mM HEPES (pH 7.3 with KOH).

For oocytes, membrane currents were recorded using the two-electrode voltage clamp by a GeneClamp 500 amplifier (Molecular Devices, Sunnyvale, CA, USA) at room temperature. Data were reproduced and analyzed with pCLAMP 10 (Molecular Devices) and Clampfit 10.2 (Molecular Devices). The bath solution contained: 90 mM KCl, 3 mM MgCl_2_, 0.15 mM niflumic acid and 5 mM HEPES (pH 7.4 with KOH). The tip resistance of glass electrodes was 0.4–1.5 MΩ when filled with the 3 M KCl pipette solution. Pilicarpine (0.001–300 μM)-induced currents and ACh (0.0001–10 μM)-induced currents were obtained by digitally subtracting the basal currents. The concentration-response curve were measured by sequential application of pilocarpine and ACh in bath solution for 30–60 s at each concentration when current amplitude reached a stable level as shown in Supplementary Figs S2 and S3. 100 μM Pilocarpine-induced current could be washed out within 1–2 min. Concentration-response curve were fitted by Hill equation with SigmaPlot 12 (Hulinks).

### Data statistics

Data statistical analysis was performed with SigmaPlot 12 (Hulinks). Results are shown as mean ± s.e.m. from *n* cells. Statistical differences between each group were evaluated by Tukey’s test. Values of *P* < 0.05 were judged statistically significant. *, ** and *** indicate values of *P* < 0.05, 0.01 and 0.001, respectively.

## Electronic supplementary material

Supplementary Information
