# Supplementary material for: Structural determinants at the M2 muscarinic receptor modulate the RGS4-GIRK response to pilocarpine by impairment of the receptor voltage sensitivity
Source: Sci Rep. 2017 Jul 21;7:6110. doi: 10.1038/s41598-017-05128-z (PMC5522400; doi:10.1038/s41598-017-05128-z)
Supplement: Supplementary file 1 — Supplementary Information [file 41598_2017_5128_MOESM1_ESM.pdf]

Supplementary Information

Structural determinants at the M2 muscarinic receptor modulate the RGS4-GIRK response to pilocarpine by impairment of the receptor voltage sensitivity

I-Shan Chen[1], Kazuharu Furutani[1,2*], Yoshihisa Kurachi[1,2*]

[1]Department of Pharmacology, Graduate School of Medicine, Osaka University
[2]Global Center for Medical Engineering and Informatics, Osaka University

Figure S1

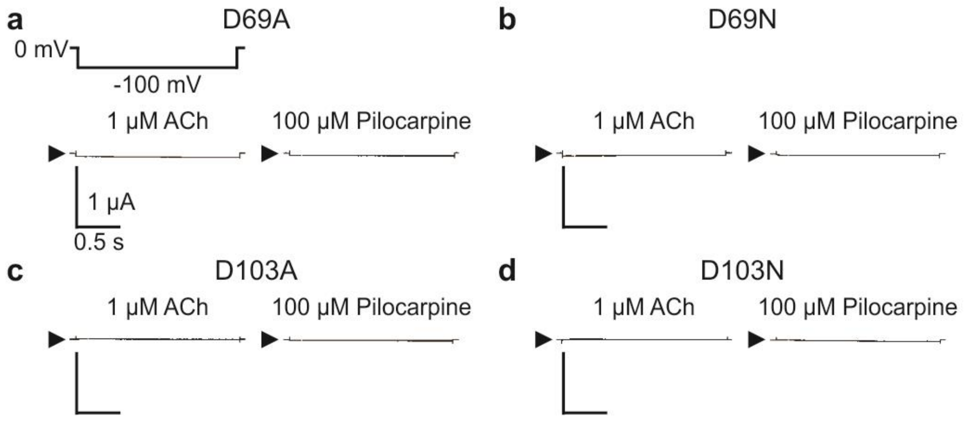

Figure S1. Effect of mutations of D69$^{2.50}$ and D103$^{3.32}$ on GIRK currents. (**a-d**) ACh (1 µM, *left panels*)- and pilocarpine (100 µM, *right panels*)-induced GIRK currents in oocytes expressing M2 muscarinic receptor mutant D69A (**a**), D69N (**b**), D103A (**c**) and D103N (**d**) were recorded at –100 mV and basal currents were subtracted. Black triangles indicate the zero current level.

Figure S2

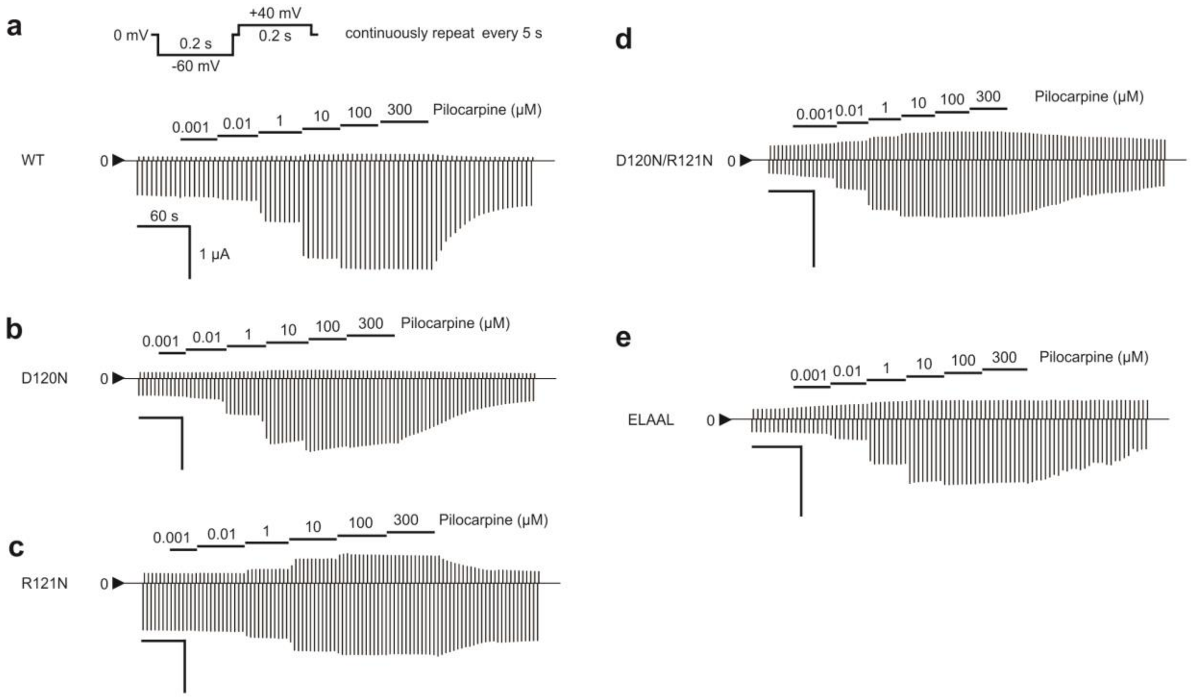

Figure S2. Concentration-dependent response of pilocarpine with WT and mutated M2 muscarinic receptor. (**a-e**) Pilocarpine (0.001 – 300 μM)-induced GIRK currents were recorded in oocytes expressing M2 muscarinic receptor WT (**a**) and mutants D120N (**b**), R121N (**c**), D120N/R121N (**d**) and ELAAL (**e**) in the presence of RGS4 with a test pulse protocol shown above continuously repeated every 5 s.

Figure S3

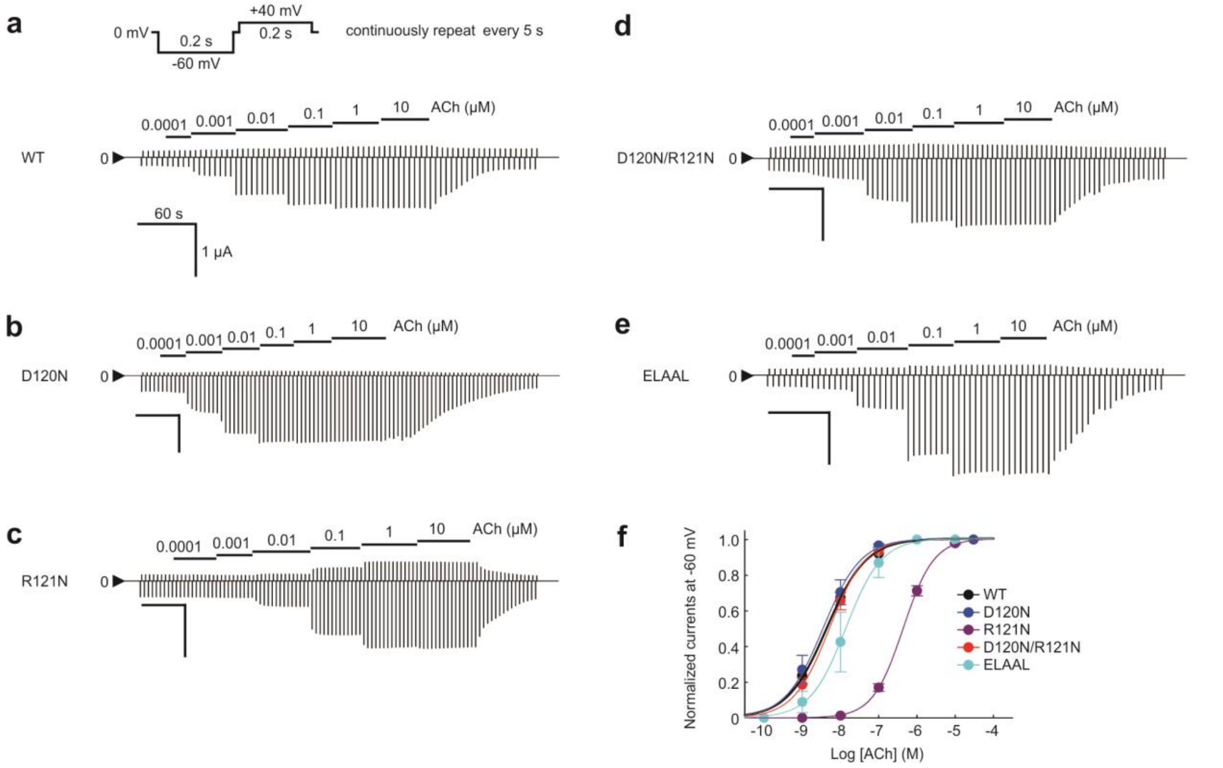

Figure S3. Concentration-response curves of ACh with WT and mutated M2 muscarinic receptor. (**a-e**) ACh (0.0001 – 10 μM)-induced GIRK currents were recorded in oocytes expressing M2 muscarinic receptor WT (**a**) and mutants D120N (**b**), R121N (**c**), D120N/R121N (**d**) and ELAAL (**e**) in the presence of RGS4 with a test pulse protocol shown above continuously repeated every 5 s. (**f**) Concentration-response curves of ACh-induced GIRK current in oocytes expressing M2 muscarinic receptor WT and mutants by fitting with the Hill equation. The maximal value of ACh-induced GIRK current was normalized to 1. The –logEC_{50} was 8.37 ± 0.03 for WT; 8.45 ± 0.03 for D120N; 6.36 ± 0.03 for R121N; 8.29 ± 0.03 for D120N/R121N; 7.86 ± 0.03 for ELAAL. Data are means ± s.e.m., n = 6 – 8.
